# Arrhythmogenic Mechanisms of Novel Biomarkers in Cardiac Electrophysiology

**DOI:** 10.1155/crp/2453934

**Published:** 2026-01-31

**Authors:** Jin Liu, Huijie Guo, Yanmin Liu, Jinchun Wu

**Affiliations:** ^1^ Qinghai University, Xining, 810007, China, qhu.edu.cn; ^2^ Department of Cardiology, Qinghai Provincial People’s Hospital, Xining, 810007, China, qhsrmyy.com

**Keywords:** arrhythmia, atrial fibrillation, biomarkers, fibrosis, inflammation, ventricular arrhythmia

## Abstract

Arrhythmia is an important cause of cardiovascular disease deaths and a serious threat to human health, but the current means of identification are limited. Biomarkers, with the advantages of easy access and rapid detection, have shown significant value in arrhythmia risk prediction, precision diagnosis, and prognosis assessment. In recent years, with the development of molecular biology and multiomics technology, some novel biomarkers have made great breakthroughs in revealing the pathological mechanisms of arrhythmia. However, clinical translation still faces challenges such as a lack of standardization of assays and insufficient clinical prospective validation. This review comprehensively searched studies published between 2010 and 2025 in PubMed, Web of Science, Embase, and CENTRAL databases, focusing on the mechanisms underlying the role of arrhythmia biomarkers in inflammation, fibrosis, autoimmunity, and electrical remodeling and clinical translation potential. Future research should focus on the combined application of multiple biomarkers and the discovery of subtype‐specific markers. Conducting large‐scale, multicenter studies to validate these biomarkers and ultimately integrate them into clinical practice is crucial for advancing biomarker‐guided individualized antiarrhythmic therapy.

## 1. Introduction

Arrhythmia refers to abnormalities in the rhythm or conduction of the heartbeat, presenting with diverse clinical manifestations, ranging from mildly insidious, asymptomatic, or occasional palpitations to severe, rapid onset, resulting in severe hemodynamic changes and lethal arrhythmias. Although arrhythmias can now be accurately diagnosed clinically by methods, such as electrocardiography or electrophysiology, early identification of high‐risk patients and prediction of malignant events are still difficult tasks. Relevant studies have shown that [1] arrhythmogenesis and alterations in the body, such as fibrosis, inflammation, and autoimmunity, can cause differential expression of blood biomarkers, and their expression levels significantly correlate with disease severity and prognosis. Therefore, clarifying arrhythmia‐related biomarkers is of great significance in revealing their pathogenesis and optimizing clinical diagnosis and treatment. Biomarkers are quantifiable indicators that can respond to pathophysiological processes, with the advantages of high sensitivity, low cost, and noninvasiveness, and are widely used in the cardiovascular field [2]. In the current context of increasing demand for precision diagnosis and treatment, identifying novel biomarkers for arrhythmias can open up new directions for precision typing and individualized treatment of arrhythmias. A prime example is the emergence of mutation‐specific precision medicine in inherited arrhythmia syndromes, which directly informs optimal therapeutic strategies [3]. This narrative review aims to summarize and critically evaluate the current landscape of arrhythmia biomarkers, discussing their underlying mechanisms and clinical evidence, and identifying key challenges and future directions for their translation into precision medicine for arrhythmia management.

## 2. Cardiac Biomarkers

### 2.1. Cardiac Troponin (cTn)

The cTn complex is a regulatory protein in myocardial contraction, and its conformational changes are regulated by calcium ion concentration [4]. Elevated cTn levels are commonly caused by ischemic cardiomyocyte injury and death through mechanisms involving cell membrane disruption, increased intracellular calcium, and cTn complex degradation and release into the serum [5]. They are commonly used in the diagnosis of myocardial infarction. Recent studies have found a significant correlation between elevated cTn levels and arrhythmic disease development and prognosis. It has been shown that protein kinase A (PKA) phosphorylation of cTnI decreases the calcium affinity of cTnC, elevated Ca^2+^ concentration activates Ca^2+^‐/calmodulin (CaM)‐dependent protein kinase II δ (CaMKIIδ), ryanodine receptor (RyR2) channels are over‐opened, and calcium spark triggers delayed afterdepolarizations (DAD), while CaMKIIδ induces early afterdepolarizations (EAD) by phosphorylating L‐type calcium channels (LTCC), inhibiting potassium currents (I_to_), and promoting late sodium currents (I_NaL_), which constitutes the main mechanism of arrhythmia‐triggered activity [6, 7]. Mice with cTnI mutations exhibit atrial matrix abnormalities, increased circulating levels of extracellular matrix remodeling and inflammatory markers, and elevated fibrosis, all of which contribute to AF development [8]. The results of a meta‐analysis showed that cTnT levels were significantly elevated in patients with new‐onset AF (SMD = 3.77, *p* < 0.001) and in patients with recurrence after AF ablation (SMD = 0.38, *p* = 0.002), whereas there was no significant correlation between cTnI and related outcomes [9]. Similar studies found that cTnI > 40 ng/L was associated with significantly higher all‐cause mortality and heart failure–related readmission rates and was an independent predictor of death (HR = 2.03, 95% CI 1.64–2.51) [10]. Combining the two studies, the clinical significance suggested by cTnT and cTnI in AF may be different, with cTnT being more sensitive to electrical remodeling or early damage to the myocardium, whereas cTnI is associated with persistent myocardial damage and structural changes. Sourour et al. found that elevated baseline cTnT significantly increased the incidence of ventricular arrhythmias (VA) in patients with implantable cardioverter‐defibrillators (ICDs), and the association persisted after adjusting for risk factors, such as coronary artery disease, heart failure, and ICD implantation status (HR = 1.61; 95% CI: 1.24–2.11) [11], but the mechanisms involved were not further explored to see whether they were based on activation of the cTn‐related pathway or myocardial fibrosis. A study by Liu et al. included 755 patients with hypertrophic cardiomyopathy (HCM) and found that cTnI ≥ 0.0265 ng/mL predicted the occurrence of nonsustained ventricular tachycardia (NSVT) (OR = 1.675, 95% CI: 1.406–1.994) [12]. Whether this association was independent of HCM needs to be further explored. Troponin is elevated in up to 46% of patients with supraventricular tachycardia (SVT), and such patients have an 11% increased risk of major adverse cardiovascular events (MACE) [13]. This elevation may stem from temporary myocardial injury caused by increased myocardial oxygen demand, or from excessive release of cTn after overstretching of myocardial fibers [14]. Although there was high heterogeneity in this study (*I* ^2^ = 93%), this result still suggests that cTn testing is useful in identifying people at high risk for SVT. Despite the value of cTn in predicting arrhythmias and adverse events, its clinical translation is still limited by ambiguous thresholds, arrhythmia specificity, and lack of clinical evidence. Future research should focus on the exploration of molecular mechanisms and the validation of targeted interventions to clarify therapeutic targets.

### 2.2. Brain Natriuretic Peptide (BNP)

BNP is a cardiac regulatory peptide synthesized primarily by ventricular myocytes, and when cardiomyocytes are exposed to stretch or other forms of mechanical stress, the three pathways of stretch‐activated G_oα_‐coupled secretion, G_qα_‐coupled secretion, and the intrinsic mechanism of selective stimulation of cytokine signaling work together to result in the secretion of BNP [15]. BNP is widely used in the diagnosis and prognosis of heart failure, and in recent years, its value in the field of cardiac arrhythmia has gradually gained attention. New research has found that BNP levels are strongly associated with arrhythmia recurrence and that elevated levels may reflect increased ventricular wall pressure [16]. The N‐terminal pro‐brain natriuretic peptide (NT‐proBNP) is cleaved from proBNP, circulates longer in the blood, and is more easily measurable. Its secretion increases when electrophysiologic alterations, such as intraventricular conduction slowing, post‐trigger depolarization, and ventricular ectopic beats, occur. Data from the LOOP study demonstrated that baseline NT‐proBNP > 125 pg/mL significantly increased the risk of AF detection. It also found that implantable cardiac monitoring reduced stroke risk by 40% in this high‐risk group [17], while showing no significant benefit in the low‐NT‐proBNP group, implying that NT‐proBNP can guide clinical decision‐making to avoid over‐screening. Analysis of the SMASH data showed that baseline NT‐proBNP concentration was associated with the risk of developing VA (HR = 1.39, 95% CI: 1.22–1.58) [18], and the association remained significant after adjustment for traditional risk factors. Electrophysiologic disturbances, such as triggered activity and refractoriness triggered by mechanical–electrical feedback during myocardial stretch, may be the mechanism by which VA occurs. A significant increase in BNP levels over time was also observed in a rat VA model established by intraperitoneal injection of BaCl_2_ solution, with differential expression detected as early as 10 min after VA [19], further supporting the association between BNP and arrhythmogenesis. Another finding was that individually blocking the endothelin‐1 (ET‐1) or TGF‐β1 signaling pathway reduced BNP concentration, but simultaneous blockade of both failed to lower BNP protein levels and was accompanied by a compensatory elevation of TGF‐β1 [19]. This provides new clues and challenges for understanding the pathophysiological mechanisms of VA and identifying intervention targets. Current studies have demonstrated the importance of BNP in the prediction of arrhythmias, but the direct pathways by which BNP causes arrhythmias remain unclear. And its specificity for predicting arrhythmias is limited by confounding factors, such as age and renal function, which has led to questions about its predictive value in this context. The prediction thresholds proposed by existing studies lack uniformity and have not been validated in multicenter cohorts, and stratification strategies for different populations have not been established.

## 3. Inflammatory Marker

Inflammation, triggered by a cascade of various leukocytes and cytokines following infection, trauma, or cellular injury, plays a crucial role in the development of arrhythmias. For electrical remodeling, cytokines released by leukocytes, such as TNF‐α, disrupt calcium homeostasis in cardiomyocytes and interfere with the function of gap junction proteins, which facilitate cell communication. For structural remodeling, TGF‐β mediates angiotensin II (Ang II) to promote fibroblast and collagen deposition, promoting cardiac hypertrophy and fibrosis. Arrhythmogenic right ventricular cardiomyopathy (ARVC) occurs when inflammation‐associated bridging granule mutations lead to myocardial necrosis and fibrosis, forming an arrhythmogenic substrate [20, 21]. The above connections provide a theoretical foundation for the exploration of inflammation and arrhythmia correlation.

### 3.1. C‐Reactive Protein (CRP)

CRP is an acute‐phase response protein synthesized by the liver and regulated by a variety of cytokines, and its elevated level reflects that the body is in an inflammatory state. CRP is not only a marker, but also a mediator that promotes and maintains inflammation. Previous studies have shown that CRP activates the Toll‐like receptor (TLR4)‐nuclear factor kappa‐B (NF‐κB) pathway, inducing the release of inflammatory factors, such as IL‐6 [22]. CRP also activates the TGF‐β1/Smad pathway, which promotes atrial fibrosis [23]. Sun et al. first demonstrated in a cardiomyocyte model that CRP drives the pathological process of AF via the TLR4/NF‐κB/TGF‐β1 axis. This was evidenced by the synchronous upregulation of TLR4, IL‐6, TGF‐β1, and p‐Smad2 expression, confirming the synchronous activation of both the TLR4/NF‐κB inflammatory axis and the TGF‐β/Smad fibrotic pathway by CRP. In addition, inhibition of NF‐κB decreased TGF‐β1 expression and Smad2 phosphorylation, demonstrating that NF‐κB positively regulates the TGF‐β/Smad pathway [24]. In patients after coronary artery bypass grafting, Olesen et al. found that CRP levels showed a dose‐dependent relationship with the incidence of postoperative atrial fibrillation (POAF). The POAF incidence was 24.5% in the CRP ≤ 90 mg/L group and 35.1% in the CRP > 175 mg/L group, a statistically significant [25], suggesting a positive correlation, but no risk factor correction was performed. Additional studies have also found high hs‐CRP levels to be associated with risk of AF development and recurrence [26, 27]. However, Corradi et al. found that pathological changes in the right auricle tissue of 239 patients were not associated with inflammatory markers (serum CRP association with fibrotic outcome *p* = 0.363) [28], a finding that seems to contradict the finding of Olesen et al. A fundamental reason for this is that Corradi et al. focused on changes occurring within the right atrium rather than the left atrium, where AF usually originates. And Olesen et al. defined POAF as atrial fibrillation in need of treatment, which is more relevant to the need for clinical intervention. The UK Biobank study confirmed that CRP was the strongest inflammatory predictor of VA and showed a linear positive correlation with VA risk. In contrast, neutrophil and monocyte counts showed a nonlinear positive correlation, while the systemic immune‐inflammation index (SII), platelet‐to‐lymphocyte ratio (PLR), and lymphocyte‐to‐monocyte ratio (LMR) showed a U‐shaped correlation [29], suggesting that different inflammatory pathways synergistically promote VA formation. However, Sourour et al.’s cohort of ICD patients showed that CRP was not able to predict VA [11], suggesting that inflammation may be the main driver of VA in the early stages of cardiovascular disease, whereas cardiac structural remodeling gradually becomes the dominant mechanism of VA as myocardial damage accumulates. Future studies need to differentiate between disease stages in the population and explore the potential value of anti‐inflammatory therapy for early arrhythmia prevention.

### 3.2. Interleukin (IL)

IL is normally transcribed and expressed in response to infection, tissue injury, or activation of immune signaling. IL‐6 promotes the production of TGF‐β1 by fibroblasts and activates its receptor, which in turn is phosphorylated by Smad to cause collagen deposition. The NLRP3 inflammasome can be activated by elevated levels of reactive oxygen species (ROS) and thioredoxin‐interacting protein (TXNIP), promoting the activation and maturation of IL‐1β and IL‐18. IL‐18 not only promotes collagen synthesis and fibrosis progression, but also synergizes with the TGF‐β1/Smad signaling pathway, exacerbating atrial structural remodeling [30, 31]. Recent studies have found that IL‐6 induces RyR2 phosphorylation through activation of the JAK‐STAT pathway, triggering calcium leakage [32, 33]. IL‐18 has been shown to activate matrix metalloproteinase (MMP‐9)–mediated extracellular matrix degradation and disrupt electrical conduction [34]. Levels of IL‐6, TNF‐α, IL‐10, and IP‐10 have been linked to nonvalvular atrial fibrillation, independently of confounding factors [35]. A study by Shitole et al. further showed that among nine inflammatory markers, including IL‐6, hs‐CRP, and leukocyte count, only IL‐6 remained significant in adjusted analyses (HR = 1.14, 95% CI: 1.07–1.21) [36], suggesting that the IL‐6 pathway was the most independent of the AF‐related inflammatory pathways. Shim et al. found that elevated levels of IL‐6 significantly activated cytokine cascade responses, such as IL‐2, IL‐8, and IL‐10 [37], which strongly supports the position of IL‐6 as an upstream regulator in the inflammatory network of AF. The study by Luan et al. was the first to identify IL‐18 as an independent inflammatory marker of AF (OR = 1.02, 95% confidence interval: 1.01–1.03), with levels increasing with the progression of AF and directly correlating with left atrial enlargement [34]. In a mouse model of sickle cell disease (SCD), Gupta et al. observed that sustained application of IL‐18‐binding protein attenuated cardiac fibrosis, decreased NF‐κB phosphorylation levels, improved diastolic function, normalized electrical remodeling, and inhibited IL‐18‐mediated ventricular tachycardia (VT) [38], which confirms that IL‐18‐targeted therapy can improve the myocardial structure, electrical remodeling, and inflammatory state. In 299 patients with new‐onset AF, IL‐34 levels and IL‐38 levels were found to be associated with an increased risk of AF‐related stroke and all‐cause mortality, respectively [39]. The IL family is strongly associated with arrhythmias, but its elevation can also be seen in noncardiogenic inflammatory conditions, such as infections and tumors, and needs to be interpreted in the clinical context. In the future, the predictive efficacy of multifactorial combinations for arrhythmias and the precise anti‐inflammatory pathways for targeted therapies can be explored.

### 3.3. Heat Shock Protein (Hsp)

In addition to underlying heart disease, arrhythmias themselves can induce electrophysiological disturbances and structural remodeling by disrupting protein homeostasis [40]. The central hub of this process is the heat shock response (HSR), where heat shock factor 1 (HSF1) is activated and binds to heat shock elements (HSEs) to upregulate the expression of genes encoding Hsp when there is an imbalance in atrial myocyte protein quality control [41]. Hsp improves cardiomyocyte calcium homeostasis, prevents the shortening of atrial action potential duration (APD) and effective refractory period, and modulates inflammation via the NF‐κB pathway. Through these mechanisms, Hsp reduces arrhythmia induction and maintenance [42]. Under physiologic conditions, cardiomyocytes express high levels of small shock proteins (HSPBs) that maintain atrial myocyte contraction and electrophysiologic function [43]. Hsp70 is an endogenous protective factor that regulates a variety of intracellular proteins and signaling pathways, enhances cellular tolerance to ischemia–reperfusion injury, and also has antiarrhythmic and antioxidant effects [44]. Geranylgeranylacetone (GGA) has been demonstrated as an HSP inducer in cellular, Drosophila, and animal models. It blocks protein homeostatic imbalance and cardiomyocyte remodeling, thereby inhibiting AF substrate formation [40]. Furthermore, GGA modulates ion channel expression alongside SERCA2a, decreasing triggered activity, APD dissociation, and AF susceptibility [45]. Van et al. observed that patients with AF recurrence within 1 week postoperatively had lower HSPB1 levels in the right atrium, with no significant difference in serum levels [46]. In a study examining the comparison between the AF nonrecurrent and recurrent groups within 7 days after mitral valve replacement, HSF1 activity was significantly elevated in the atrial tissue of patients in the nonrecurrent group, accompanied by an upregulation of the expression of Hsp70 and Hsp27 [47], confirming the cardioprotective role of the HSP family. However, recent clinical studies have confirmed that serum HSP27, HSP70, and HSP60 levels are not statistically different between different AF types and cannot be used as biomarkers for staged diagnosis of AF or prediction of recurrence. In contrast, HSP27 levels in serum samples from patients with recurrent AF increased within one year after pulmonary vein isolation [48], suggesting that HSP27 levels can be used as a postablation monitoring indicator. The above studies suggest that Hsp may play an antifibrotic cardioprotective role in atrial tissue, which is negatively associated with adverse outcomes, whereas in circulating serum, it is used as a marker of injury, which is positively associated with recurrence after ablation. Future therapies targeting HSPs need to precisely differentiate the mechanism of action in different tissues, and biomarker exploration should focus on tissue‐specific assays.

### 3.4. Somatostatin Receptor Type‐2 (sST2)

ST2 is a member of the IL‐1 receptor family, and its ligand IL‐33 binds to membrane‐bound ST2L to activate cardioprotective pathways. In experimental models, IL‐33 and ST2L signaling pathways have the ability to attenuate myocardial fibrosis, inhibit cardiomyocyte hypertrophy, and improve myocardial function [49]. Under mechanical stress and volume loading of cardiomyocytes or myocardial fibers, sST2 is secreted in large quantities. sST2 induces cardiac dysfunction by competitively binding to IL‐33, effectively blocking the cardioprotective effects of the IL‐33/ST2L signaling pathway, in addition to promoting myocardial fibrosis and pathological remodeling through the activation of inflammation‐related pathways [50]. As an important biomarker of cardiac stress and fibrosis, sST2 has demonstrated unique value in the prediction and management of cardiac arrhythmias. Elevated sST2 triggers fibrosis, which in turn disrupts myocardial electrical conduction continuity and increases heterogeneity; affects ion channels and prolongs APD; and creates conduction blocks and slow conduction pathways that provide the basis for refractory arrhythmias while generating triggered activity. Evidence from clinical studies further supports the association between sST2 and arrhythmias. A meta‐analysis of data showed that higher sST2 levels were significantly associated with the risk of AF occurrence (HR = 1.04, 95% CI: 1.02–1.07), recurrence after ablation (HR = 1.09, 95% CI: 1.02–1.16), and the occurrence of adverse cardiovascular events following AF (HR = 1.60, 95% CI: 1.13–2.27) [51]. Melchor et al. found sST2 to be a useful biomarker for predicting AF recurrence after electrical cardioversion [52]. In patients with ARVC, plasma sST2 levels were significantly higher in those who developed VA than in those who did not, and this association persisted after correction for right ventricular function [53]. This study also found that sST2 correlated with left ventricular function [53], confirming that the role of sST2 as a biomarker of cardiac stress, inflammation, and fibrosis is not limited to the atria. sST2 is clinically stable, less susceptible to other confounding factors, and directly correlates with fibrosis, providing clear value in predicting and assessing the risk of AF and VA. Future studies should delve deeper into the role of sST2 in specific arrhythmia mechanisms and assess the potential of targeting the IL‐33/ST2 pathway to exert antifibrotic effects and reduce arrhythmogenesis in animal models, providing new ideas for future therapies.

### 3.5. Others

ROS are widely present in human cells and play an important role in information transfer and apoptosis [54]. Mitochondria produce excess ROS in response to certain stimuli and cause arrhythmias by affecting the activity of Ca^2+^‐associated proteins, mitochondrial permeability transition pore (mPTP) components, connexin 43 (Cx43), hyperpolarization‐activated cyclic nucleotide‐gated potassium channel 4 (HCN4), and other ion channels [55]. Selective blockade of this pathway has emerged as a new therapeutic target to prevent arrhythmogenesis. The pathophysiological link between inflammatory mechanisms and arrhythmias has been recognized for several years. Through concerted efforts, some inflammatory markers have been recognized as predictors of arrhythmia. However, they have not yet been effectively translated into clinical practice. Therefore, continued investigation is needed to discover more accurate biological factors for arrhythmia identification and to support their use with more rigorous data.

## 4. Autoantibody Biomarkers

In recent years, the pathogenic role of autoimmune‐related markers in the cardiovascular system has been demonstrated [56]. Rheumatoid arthritis (RA) has been reported to increase the risk of atrial fibrillation (OR = 1.060; 95% CI: 1.028–1.092) [57] and to predict the risk of stroke after AF [58]. Genetic studies further revealed that knockdown of the TMEM45A gene, which is aberrantly expressed in SLE patients, suppressed the development of AF and atrial fibrosis. All of these results suggest that arrhythmias are closely related to autoimmunity. Sun et al. found that anti‐β1‐adrenergic receptor autoantibodies (β1‐AAb) triggered calcium disturbances in atrial myocytes through activation of the adrenergic receptor (β1AR)–driven CaMKII/RyR2 pathway and drove phenotypic transformation of fibroblasts to myofibroblasts, exacerbating electrical instability and structural remodeling [59]. Similar studies found that anti‐β1‐R levels were the strongest independent predictor of NVAF, followed by anti‐M2‐R and IL‐6 [60], suggesting that autoimmunity and inflammation are synergistically involved in the development of AF. Although this study confirmed the independent predictive value of autoimmune markers and inflammatory factors in NVAF, the predictive efficacy of joint modeling of the two was not further evaluated, and the mechanism of their role in arrhythmias warrants further investigation. Notably, sudden cardiac death (SCD) is autopsy‐negative in approximately 15% of cases, of which ≤ 30% are attributable to inherited ion channelopathies [61], and acquired autoimmune mechanisms are equally critical to the impact of ion channels. Beta‐adrenergic stimulation leads to ion channel remodeling, characterized by adrenergic‐enhanced increases in inward L‐type calcium currents (I_ca, L_) and fast‐activating delayed rectifier K^+^ currents (I_kr_), alongside decreases in slow‐activating delayed rectifier potassium currents (I_ks_). These changes result in abnormally prolonged cardiac repolarization and the formation of EADs, creating a substrate conducive to lethal arrhythmias [62]. In addition, fetal cardiac tests have confirmed that anti‐Ro/SSA antibodies can be transferred across the placenta as early as 11 weeks of gestation, cross‐reacting with LTCC or T‐type calcium channels and inhibiting calcium currents, leading to abnormalities in the sinus node and atrioventricular conduction, and that anti‐Ro/SSA antibodies can down‐regulate the expression of LTCC, triggering apoptotic cell death and leading to fibrosis [61]. Although the above studies confirm the predictive value of serum antibodies for arrhythmias, the lack of large clinical data to support them, insufficient standardization, and the lack of evidence for interventions all pose significant challenges to clinical translation.

## 5. Fibrosis‐Related Biomarkers

Relevant studies have shown that remodeling leads to the proliferation of fibroblasts and the production of extracellular matrix. When a certain threshold is reached, rhythmic disturbances in cardiac electrical activity occur, characterized by increased myocardial autoregulation, interference with ion channels, obstruction of action potential conduction, and ultimately, arrhythmias [63].

### 5.1. Galectin‐3 (Gal‐3)

Elevated Gal‐3 levels increase the inflammatory response and promote the progression of fibrosis, leading to cardiac remodeling and fibrotic arrhythmogenic effects [64]. Basic research revealed that Gal‐3 binding to its membrane surface receptor CD98 significantly shortened the APD of atrial myocytes and induced calcium dysregulation; molecular analysis further revealed that the Gal‐3/CD98 axis activated the CaMKII/RyR2 pathway, forming the basis of pro‐arrhythmic cellular electrical activity. In a mouse model, systemic administration of Gal‐3 significantly increased the incidence of atrial premature beats and activated the NF‐κB/NLRP3 inflammasome signaling pathway, which drove inflammatory infiltration and collagen deposition in atrial tissue [65]. Conversely, functional blockade of CD98 simultaneously reversed these changes, confirming that targeted intervention of the Gal‐3/CD98 signaling pathway may provide a novel therapeutic strategy for patients with high Gal‐3 expression in AF. Gal‐3 and TGF‐β1 regulate each other in the development of AF and synergistically promote downstream Smad2 phosphorylation and collagen I deposition [66], which together constitute the central hub of the profibrotic signaling network. Clinical data showed that patients with elevated serum Gal‐3 levels 6 months after AF ablation had an elevated risk of AF recurrence (HR = 2.91), whereas baseline serum Gal‐3 levels did not correlate with either the degree of left auricle fibrosis or left atrial Gal‐3 [67], suggesting that serum Gal‐3 does not correlate with localized atrial fibrosis, but reflects the systemic fibrosis status. Pietrzak et al. found that Gal‐3 plasma concentrations were significantly higher in adolescent VA patients (13.45 ± 11.4 ng/mL) than in healthy controls, with a moderate positive correlation between ventricular diameter z‐scores (*r* = 0.47) and a moderate negative correlation between cardiac magnetic resonance ejection fraction (*r* = −0.49) [68]. Gal‐3 not only reflects the fibrosis status but also directly correlates with ventricular remodeling and dysfunction in adolescent VA patients, and can be used as a biological marker for early identification of myocardial structural abnormalities.

Based on the above studies, there is a correlation between elevated Gal‐3 concentration and increased risk of arrhythmia, but Gal‐3 as a biomarker for arrhythmia is still at an early stage, and there is insufficient evidence from the current studies. Some studies showed only a moderate correlation between Gal‐3 and related cardiac function indexes. Furthermore, there is a lack of established clinical thresholds and validation regarding the safety of Gal‐3‐targeted therapies in humans.

### 5.2. Growth Differentiation Factor 15 (GDF‐15)

GDF‐15 is associated with inflammatory states in many tissues and is a stress protein whose expression level in the heart is significantly elevated in tissue injury and inflammatory states [69]. The expression of GDF‐15 is regulated by several transcription factors and is secreted in an inactive manner. After being secreted, GDF‐15 exerts its function through autocrine or paracrine mechanisms and via two intracellular signaling pathways: Smad protein‐dependent and Smad protein‐independent. GDF‐15 was identified as an independent prognostic marker for severe bleeding and death in AF in the ARISTOTLE study [70] and was included in the ABC bleeding score [71]. Recent studies have shown that GDF‐15 > 767.5 pg/mL was an independent risk predictor of significant left atrial fibrosis (OR = 3.318, 95%:1.184–9.298) [72]. The combination of GDF‐15 > 767.5 pg/mL and the left atrial volume index (LAVI) had moderate power for predicting left atrial fibrosis (AUC = 0.762). However, GDF‐15 has no predictive value for the development of either AF or VA [11, 73, 74]. GDF‐15, which cannot be used as a biomarker to predict the occurrence of arrhythmias, currently has clear clinical value in identifying patients with severe bleeding and death, and its detection in the clinic may assist in assessing the degree of fibrosis and the risk of anticoagulated bleeding.

## 6. RNA

### 6.1. MicroRNAs (miRNAs)

The involvement of miRNAs in electrophysiological and structural remodeling explains their possible role in arrhythmogenesis [75]. MiR‐21 and its downstream target Spry1 participate in structural remodeling and regulate the STAT3 pathway, thereby mediating inflammation‐associated fibrosis. MiR‐21 also serves as a key regulator of TGF‐β‐induced endothelial‐to‐mesenchymal cell transition via the PTEN/AKT‐dependent pathway. Circulating miR‐21 levels reflect fibrotic changes contributing to the arrhythmogenic substrate in AF events [76]. Another study found that miR‐21, miR‐133b, and miR‐499 were directly associated with the development of AF and involved in apoptosis and fibrosis [77]. Compared with traditional biomarkers (e.g., BNP or cTn), miRNAs do not yet have strong evidence for predicting arrhythmias, and the assay technology is complex and costly, but previous studies provide direction for the medical community to continue exploring the potential role of miRNAs in cardiac arrhythmias.

### 6.2. Long Noncoding RNA (lncRNA)

lncRNAs are a class of noncoding RNA molecules exceeding 200 nucleotides in length. In recent years, studies have revealed that lncRNAs are differentially expressed in the cardiac tissues of patients with AF and that they regulate ion channel expression, calcium homeostasis, myocardial fibrosis, and signaling pathway interactions through multiple mechanisms [78]. Post‐transcriptional gene regulation by lncRNAs may contribute to AF progression [79]. A study by Wang et al. found that silencing a specific lncRNA effectively blocked the TGF‐β/Smad pathway and inhibited fibrosis [80], providing a new strategy to prevent structural remodeling in AF. The regulatory role of lncRNAs in gene expression may play a key role in arrhythmogenesis, but this area remains poorly understood and has not yet been translated into widespread clinical practice.

## 7. Phosphorylation

Protein phosphorylation is a crucial post‐translational modification involved in regulating diverse physiological processes and plays an important role in cardiac pathophysiology. Protein kinases and phosphatases control the phosphorylation and dephosphorylation of a wide range of cardiac proteins, thereby fine‐tuning cardiac electrophysiology and function, and are involved in the development and maintenance of arrhythmias by affecting the activity of ion channels, structural proteins, and signaling pathways [81]. CaM mutants associated with long QT syndrome (LQTS) act as ion channel regulators, affect phosphorylation of CaMKIIδ, and represent an important molecular mechanism contributing to LQTS‐related arrhythmias [82]. Deficiency of mitogen‐activated protein kinase kinase‐7 (MKK‐7) impairs the phosphorylation of histone deacetylase‐2 (HDAC‐2), thereby reducing transcription of potassium channel genes, resulting in delayed repolarization and VA [83]. Research on the relationship between phosphorylation and arrhythmias is ongoing and continues to deepen. With the emergence of new technologies, the role of phosphorylation in predicting and treating arrhythmia is expected to expand.

## 8. Trace Element

Electrolyte disturbances are known to cause arrhythmias [84, 85]: Abnormal K^+^ concentrations alter the excitability of cardiomyocytes, leading to rhythm disorders. Mg^2+^ exerts antiarrhythmic effects by modulating cardiomyocyte excitability through inhibition of Ca^2+^ entry, and Ca^2+^ and H^+^ can lead to life‐threatening LQTS through voltage‐dependent blockade of the cardiac potassium channel hERG. Heavy metals can promote the production of ROS, further promoting oxidative stress and inducing inflammation. This leads to endothelial dysfunction, abnormal lipid metabolism, disruption of ionic homeostasis, and epigenetic changes [86]. Selenium is a trace element with antioxidant properties that prevents the formation of ROS [87], maintains normal electrical activity of the heart, inhibits atrial structural remodeling [88], and may prevent AF by inhibiting oxidative stress mechanisms; therefore, selenium deficiency may be a reliable signal for the development of AF. The zinc transporter protein (ZIP8) and PEP1‐Cu/Zn superoxide dismutase (PEP‐1‐SOD1) are also closely linked to arrhythmias [89, 90]. Currently, most studies focus on the association between a single element and arrhythmia. Further research should explore the combined testing of multiple trace elements to characterize their alteration patterns across different arrhythmia types.

## 9. Discussion

With the rapid development of modern medical technology, the level of cardiovascular diagnostic and treatment technology has improved dramatically. However, arrhythmia remains a leading cause of cardiovascular mortality and morbidity. Current interventions, such as catheter ablation and ICDs, though effective, are often employed after arrhythmia establishment and face challenges, such as recurrence and procedural complications. This underscores the pressing need for biomarkers that can improve early risk stratification and guide personalized management.

**Figure 1:**
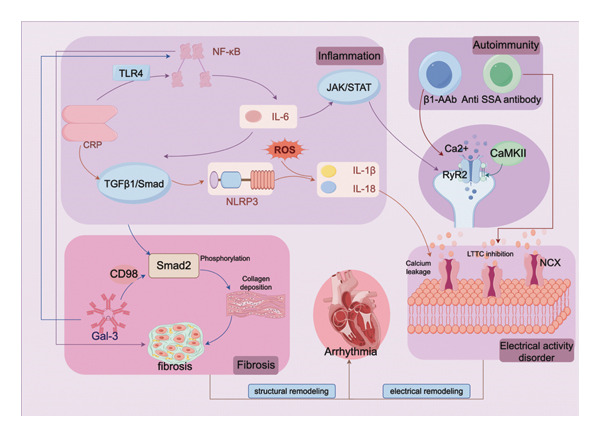
Inflammation–autoimmunity–fibrosis–electrical remodeling interactions. Figure Legend: This schematic outlines the core arrhythmogenic pathways. Inflammatory stimuli activate NF‐κB and JAK/STAT signaling, while also driving fibrosis through the TGF‐β1/Smad and Gal‐3/CD98 pathways. At the same time, autoimmunity and inflammation act together to induce electrical remodeling by activating CaMKII, triggering RyR2 phosphorylation and calcium leakage, thereby promoting triggered activity. The resulting fibrotic substrate synergizes with cardiomyocyte electrical instability to establish the substrate for both atrial and ventricular arrhythmias. Abbreviations: CRP, C‐reactive protein; TLR4, Toll‐like receptor 4; IL, interleukin; ROS, reactive oxygen species; NLRP3, NLRP3 inflammasome; Gal‐3, galectin‐3; β1‐AAb, anti‐β1‐adrenergic receptor autoantibodies; CaMKII, calcium‐/calmodulin‐dependent protein kinase II; RyR2, ryanodine receptor 2; LTCC, L‐type calcium channel; NCX, sodium–calcium exchanger.

These biomarkers reflect underlying mechanisms of arrhythmia through fibrosis, inflammation, autoimmunity, phosphorylation, and molecular proteins (the interactions between inflammation, autoimmunity, fibrosis, and electrical remodeling are shown in Figure 1), and their potential role in improving early arrhythmia detection and risk stratification is discussed (a summary of key arrhythmia biomarkers is provided in Table 1). Among the biomarkers discussed, BNP/NT‐proBNP and sST2 currently demonstrate the most reliable and translatable predictive potential for arrhythmias. Although BNP/NT‐proBNP has long been applied in the field of heart failure, its evidence for predicting AF and VA is equally robust, and large cohort studies, such as LOOP and SMASH, have confirmed its value in guiding clinical decision‐making [17, 18]. In contrast, although cTn shows a significant association with arrhythmia risk, its lack of arrhythmia specificity limits its primary value to general prognostic assessment rather than specific arrhythmia diagnosis. sST2 stands out due to its direct association with cardiac fibrosis, clinical stability, and consistent confirmation across multiple studies as an independent predictor of AF incidence, recurrence, and VA [51–53].

**Table 1:** Arrhythmia biomarkers at a glance.

Biomarker type	Mechanism link to arrhythmia	Clinical status	References
cTn	Disruption of calcium homeostasis via CaMKIIδ/RyR2 pathway, triggering DADs/EADs; promotion of inflammation and fibrosis via cTnI mutations.	Associated with new‐onset AF, AF recurrence, VA, and adverse prognosis. Established in MI; emerging evidence for arrhythmia risk stratification.	[5–8]
BNP/NT‐proBNP	Secretion induced by ventricular wall stress and electrophysiological alterations; interacts with profibrotic pathways	Predicts the occurrence and recurrence of AF and VA. Well‐established in heart failure; strong evidence for AF/VA prediction, but influenced by age/renal function.	[16–19]
Inflammation (CRP)	Activates TLR4/NF‐κB axis, disrupting calcium handling and gap junctions; drives collagen deposition via TGF‐β1/Smad pathway.	Positively correlated with risk of postoperative AF (POAF) and VA in the general population. Strong epidemiological association; limited by low specificity and controversial predictive value	[23, 24]
Inflammation (IL‐6 and IL‐18)	IL‐6 induces calcium leak via JAK‐STAT/RyR2; IL‐18 disrupts electrical conduction via MMP‐9; both synergistically promote collagen deposition and structural remodeling.	Independent predictors of AF; levels correlate with AF progression and left atrial enlargement. Promising; specificity is limited; targeted therapies under investigation.	[30–38]
Hsp	Improves calcium homeostasis and prevents APD shortening; exerts anti‐inflammatory and antifibrotic effects via NF‐κB pathway.	Tissue Hsp: Cardioprotective, associated with favorable outcomes. Serum Hsp: Potential marker of injury/ablation recurrence. Experimental; dual role in tissue vs. serum complicates clinical interpretation.	[40, 42–45]
sST2	Promotes fibrosis, conduction heterogeneity, APD prolongation, and conduction block; blocks protective IL‐33/ST2L signaling and activates profibrotic pathways.	Predicts AF occurrence/ablation recurrence, VA, and adverse cardiovascular events. Promising; clinically stable, directly correlated with fibrosis, less affected by confounders.	[49, 50]
ROS	Affects the activity of calcium‐handling proteins, gap junctions, and ion channels, leading to electrophysiological instability. Causes electrophysiological disturbances and arrhythmias.	Early research; the ROS pathway is a potential therapeutic target, but lacks biomarker validation.	[54, 55]
Autoimmunity	Causes calcium dysregulation, inhibits ion channel function, causes conduction abnormalities; drives fibroblast transformation and apoptosis.	Autoimmune diseases increase AF risk; autoantibodies are strong independent predictors of NVAF. Promising significant challenges in standardization, large‐scale data, and interventional evidence.	[59–63]
Fibrosis (Gal‐3)	Activates CaMKII/RyR2 via CD98 binding, causing calcium dysregulation and triggered activity; core hub in profibrotic network.	Associated with AF recurrence postablation and ventricular remodeling and dysfunction in adolescent VA. Early research stage; serum levels may reflect systemic fibrosis; lacks standardized thresholds and therapeutic validation.	[65–68]
Fibrosis (GDF‐15)	Stress‐responsive cytokine elevated in tissue injury and inflammation; involved in fibrotic processes via Smad‐dependent and Smad‐independent pathways.	Not predictive of arrhythmia onset; independent predictor of significant left atrial fibrosis. Clear clinical value for assessing bleeding risk and mortality in AF, not for predicting arrhythmias.	[70–72]
RNA (miRNAs)	Regulates ion channel expression and calcium homeostasis; promotes fibrosis via pathways, such as STAT3 and TGF‐β/PTEN/AKT	Circulating levels are associated with AF development and the fibrotic substrate. Predominantly experimental; complex and costly assays; insufficient predictive evidence for clinical use.	[76–78]
RNA (lncRNAs)	Regulates ion channel expression and calcium homeostasis; modulates fibrotic processes via pathways, such as TGF‐β/Smad. Differentially expressed in AF cardiac tissue; post‐transcriptional regulation contributes to AF progression.	Early research stage; limited mechanistic understanding; not yet clinically applicable.	[79–81]
Phosphorylation	Signaling protein phosphorylation fine‐tunes cardiac electrophysiology by regulating ion channels; central to signaling. Involved in the development and maintenance of arrhythmias through effects on electrical activity and signaling.	Extensive basic research; high potential as a therapeutic target; predictive role to be fully utilized.	[82–84]
Trace element	Directly affects ion channels and excitability; heavy metals induce ROS/oxidative stress, while selenium has antioxidant effects. Electrolyte disturbances directly cause arrhythmias; trace element imbalance is linked to AF risk.	Research stage; future direction lies in multi‐element panel testing to identify characteristic profiles.	[87–90], [91]

*Note:* CaMKIIδ, calcium‐/calmodulin‐dependent protein kinase IIδ; DADs, delayed afterdepolarizations; EADs, early afterdepolarizations; HDAC2, histone deacetylase 2; IL, interleukin; MKK‐7, mitogen‐activated protein kinase kinase 7; NF‐κB, nuclear factor kappa‐light‐chain‐enhancer of activated B cells; NLRP3, NLRP3 inflammasome; ST2L, membrane‐bound ST2; NT‐proBNP, N‐terminal pro‐B‐type natriuretic peptide; cTn, cardiac troponin; cTnI, cardiac troponin I; BNP, B‐type natriuretic peptide; miRNAs, microRNAs; MMP‐9, matrix metalloproteinase‐9; RyR2, ryanodine receptor 2.

Abbreviations: AF, atrial fibrillation; APD, action potential duration; CRP, C‐reactive protein; Gal‐3, galectin‐3; GDF‐15, growth differentiation factor 15; Hsp, heat shock protein; LQTS, long QT syndrome; MI, myocardial infarction; NVAF, nonvalvular atrial fibrillation; POAF, postoperative atrial fibrillation; ROS, reactive oxygen species; sST2, soluble suppression of tumorigenicity 2; TGF‐β1, transforming growth factor beta 1; TLR4, Toll‐like receptor 4; VA, ventricular arrhythmias.

The translational application of novel biomarkers faces significant obstacles due to several major limitations. First, for most emerging biomarkers, such as lectin‐3 and leukocyte IL, standardized testing methods and universally accepted diagnostic thresholds are generally lacking. Second, the specificity of individual biomarkers is often low; for example, elevated levels of CRP and IL‐6 are observed in various noncardiac inflammatory diseases, making it difficult to attribute their elevation solely to arrhythmogenic pathophysiology. Third, promising biomarkers, such as autoantibodies and RNA markers, derive their clinical evidence from small‐ to medium‐sized single‐center observational studies, lacking validation from large‐scale, multicenter, prospective cohort studies. Furthermore, the causal role of many biomarkers in arrhythmia development remains largely unproven.

Future research must shift toward more comprehensive and rigorous methodologies. The immediate priority is to conduct large‐scale, prospective, multicenter studies to validate the most promising candidate biomarkers. Integrating multiomics data is essential for discovering novel biomarkers and elucidating complex pathophysiological networks. Leveraging artificial intelligence to analyze these massive datasets holds the potential to unlock the power of multibiomarker combinations. Ultimately, biomarker‐guided therapy remains the true benchmark for clinical utility.

## Ethics Statement

The authors have nothing to report.

## Disclosure

All authors read and approved the final manuscript and approved the final version to be published.

## Conflicts of Interest

The authors declare no conflicts of interest.

## Author Contributions

This study was designed by Yanmin Liu and Jinchun Wu, and Jin Liu wrote the manuscript. Yanmin Liu and Jinchun Wu reviewed and edited the manuscript and contributed to the discussion.

## Funding

This work was supported by the Mechanism of Mild Hyperbaric Oxygen Therapy in High‐Altitude Pulmonary Hypertension (Project No.: 2022‐ZJ‐756), 2022, Qinghai Province “Kunlun Talent—High‐End Innovation and Entrepreneurship Talent” Project (No project number), 2025, Key Project of the Qinghai Provincial Health System (Project No.: 2025‐wjzd‐01).

## Data Availability

The authors have nothing to report.
